# Ovotoxic Effects of Galactose Involve Attenuation of Follicle-Stimulating Hormone Bioactivity and Up-Regulation of Granulosa Cell p53 Expression

**DOI:** 10.1371/journal.pone.0030709

**Published:** 2012-02-02

**Authors:** Sayani Banerjee, Pratip Chakraborty, Piyali Saha, Soma Aditya Bandyopadhyay, Sutapa Banerjee, Syed N. Kabir

**Affiliations:** 1 Reproductive Biology Research, CSIR-Indian Institute of Chemical Biology, Jadavpur, Kolkata, West Bengal, India; 2 Department of Infertility, Institute of Reproductive Medicine, Salt Lake City, Kolkata, India; Florida International University, United States of America

## Abstract

Clinical evidence suggests an association between galactosaemia and premature ovarian insufficiency (POI); however, the mechanism still remains unresolved. Experimental galactose toxicity in rats produces an array of ovarian dysfunction including ovarian development with deficient follicular reserve and follicular resistance to gonadotrophins that characterize the basic tenets of human POI. The present investigation explores if galactose toxicity in rats attenuates the bioactivity of gonadotrophins or interferes with their receptor competency, and accelerates the rate of follicular atresia. Pregnant rats were fed isocaloric food-pellets supplemented with or without 35% D-galactose from day-3 of gestation and continuing through weaning of the litters. The 35-day old female litters were autopsied. Serum galactose-binding capacity, galactosyltransferase (GalTase) activity, and bioactivity of FSH and LH together with their receptor competency were assessed. Ovarian follicular atresia was evaluated in situ by TUNEL. The *in vitro* effects of galactose were studied in isolated whole follicles in respect of generation of reactive oxygen species (ROS) and expression of caspase 3, and in isolated granulosa cells in respect of mitochondrial membrane potential, expression of p53, and apoptosis. The rats prenatally exposed to galactose exhibited significantly decreased serum GalTase activity and greater degree of galactose-incorporation capacity of sera proteins. LH biopotency and LH-FSH receptor competency were comparable between the control and study population, but the latter group showed significantly attenuated FSH bioactivity and increased rate of follicular atresia. In culture, galactose increased follicular generation of ROS and expression of caspase 3. In isolated granulosa cells, galactose disrupted mitochondrial membrane potential, stimulated p53 expression, and induced apoptosis in vitro; however co-treatment with either FSH or estradiol significantly prevented galactose-induced granulosa cell p53 expression. We conclude that the ovotoxic effects of galactose involves attenuation of FSH bioactivity that renders the ovary resistant to gonadotrophins leading to increased granulosa cell expression of p53 and follicular atresia.

## Introduction

Premature ovarian failure, currently referred to as premature ovarian insufficiency (POI), is a frequent finding in women with galactosaemia [1]–[3]. Galactosaemia, an inherited inborn error of the major galactose assimilation pathway caused by galactose-1-phosphate uridyltransferase (GALT) deficiency, produces wide phenotypes of ovarian dysfunction [4]. The prevalence of POI in galactosaemic population is 1 in 10,000 for women between 15 and 29 years of age, and 7.6 in 10,000 for women aging between 30 to 39 [5]. In some women ovarian failure is a consequence of premature depletion of follicular reserve (afollicular or follicle depletion type of POI), while the other galactosaemic women do exhibit the presence of follicles that are refractory to gonadotrophin stimulation and therefore suffer from arrested growth and maturation (follicle dysfunction type of POI or resistant ovary syndrome) [6]. Despite more than four decades of intense research, the cause and effect relationships between galactosaemia and POI, and the molecular mechanisms of galactose toxicity remain elusive; however, the general consensus is that the ovarian pathology is the aftermath of toxic effects of galactose and its metabolites both at the ovarian and extra-ovarian levels [7]–[9]. Rodents placed on high galactose diet provide an excellent model for galactose toxicity [10]–[12]. We have earlier demonstrated that experimental galactose toxicity in rats produced an array of ovarian dysfunctions that characterize the basic tenets of diverse phenotypes of POI [13]. Embryos exposed to high galactose *in utero* suffer from significant attenuation of germ cell migration and develop ovaries with deficient follicular reserve [14]. Liu *et al.* [15] reported that high galactose diet down regulated the oocyte-specific growth factor, GDF-9, which is obligatory for folliculogenesis, and inhibition of follicular development was a secondary consequence. Lai *et al.* [16] demonstrated that immature rats fed with high galactose diet exhibited higher expression of Fas and Fas-ligand but lower expression of Xiap and Riap, suggestive of increased apoptotic damage of the ovary. These mechanisms, however, could not explain the full spectrum of ovarian dysfunction since normal adult mice with optimum follicular reserve also exhibited ovarian failure in the form of follicular resistance to gonadotrophins following exposure to high galactose [17]. Thus the precise mechanisms underlying ovotoxic effects of galactose is still far from clear.

We have demonstrated that galactose toxicity renders the ovary refractory to gonadotrophins; but suppression of endogenous gonadotrophins by GnRH receptor down regulation improves ovarian response to exogenous gonadotrophins [13]. This observation questions if the gonadotrophins produced under galactose toxicity do possess normal bioactivity.

Reports indicate that galactosaemia interferes with the galactosylation process. An abnormal glycosylation pattern has been documented in some galactosaemic females with partial absence of terminal disaccharides leading to synthesis of the neutral isoform of FSH [18]. *In vitro* and *in vivo* experiments have shown that deglycosylated FSH has a higher binding affinity to its receptor than the glycosylated form, but is unable to activate the second messenger system [19]. Despite a growing body of evidence holding glycosylation defect and consequent loss of gonadotrophin biopotency as the major causes of ovarian resistance to gonadotrophins, the production of aberrant FSH isoform with reduced bioactivity has been recently contradicted [20]–[21]. In sheep, high doses of galactose inhibited FSH-induced differentiation of granulosa cells cultured *in vitro* [7]. Fraser *et al.* [22] suggested that an acquired anomaly of gonadotrophin receptors perhaps attributes to the process. However, no direct evidence in support of anomaly of gonadotrophin receptors is available so far. Thus, the mechanism underlying the ovotoxic effects of galactose remains unresolved.

The present investigation explores the possibility of galactosylation defect of gonadotrophins and seeks evidence if galactose toxicity impairs bioactivity of gonadotrophins or their receptor competency. It also addresses the effect of galactose on follicular atresia and granulosa cell apoptosis.

## Results

### Effects of galactose *in vivo*

#### Serum capacity to bind galactose

In the presence of purified GalTase, serum proteins of the galactose-exposed rats exhibited significantly higher (*P* = 0.007) incorporation of radiolabeled galactose (fmol/µg serum protein) as compared to the serum proteins from controls (galactose-exposed: 1.32±0.02 *vs.* control: 1.09±0.07).

#### Serum galactosyltransferase (GalTase) activity

Serum GalTase activity was assessed in respect of the capacity of serum to catalyse the transfer of radiolabeled galactose from UDP-galactose to ovalbumin (endogenous serum protein that also served as galactose acceptor was not taken into account). The overall catalytic transfer of radiolabeled galactose (fmol/µg protein) from UDP-galactose to ovalbumin was significantly lower (*P* = 0.0003) in the presence of study sera (4.52±0.32) as compared to that under the influence of control sera (6.49±0.30).

#### Serum gonadotrophic activity

**Figure 1:**
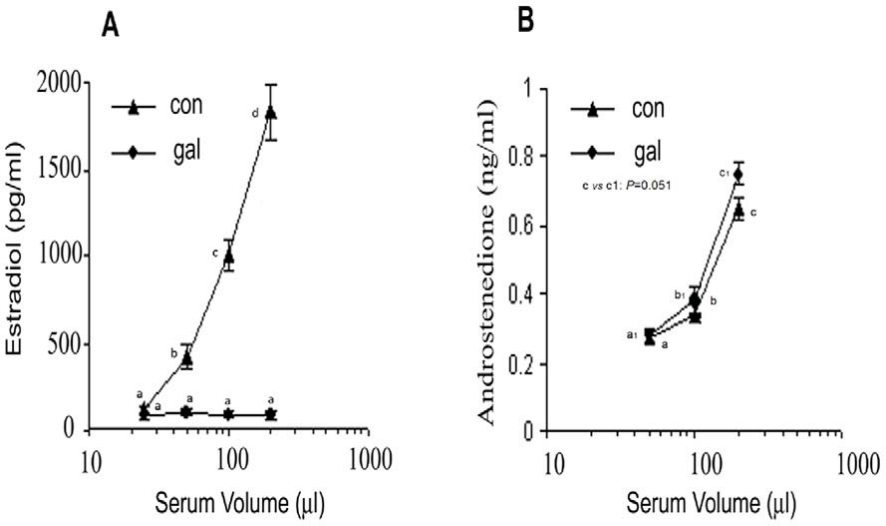
Follicular cell steroidogenic response to serum gonadotrophins. Granulosa and theca cells retrieved from ovaries of control rat were cultured in the presence of both control and galactose-exposed rat sera. Serum FSH-like activity was evaluated with respect to granulosa cell production of E_2_ (Fig. 1A), while theca cell production of androstenedione (Fig. 1B) was the measure of serum LH-like activity. Values are expressed as mean ± SEM of 4 determinations in each datum point. In Fig. 1A, the data points with different letters (a, b, c, d) differ significantly (*P*<0.0001). In Fig. 1B, androstenedione production under the influence of 200 µl study serum was comparatively higher than that of control sera but the difference does not reach the significance level.

Production of estradiol (E_2_) by the cultured control granulosa cells under the influence of sera from control and study population has been presented in Figure 1A. E_2_ production, a measure of serum FSH-like activity, increased significantly (P<0.0001) over the basal level almost in a linear fashion in response to an increase in the volume of control sera between 0.025–0.200 ml. By contrast, addition of same volumes of study sera was not rewarded by any increase in E_2_ production over the basal level, suggesting attenuated serum FSH-like activity of the galactose-exposed rats.

Figure 1B presents theca cell production of androstenedione in response to different volumes of control and experimental sera that served as the source of LH. Androstenedione production under the influence of both control and study sera gradually increased over the basal level, that were comparable between the groups. The steroidogenic response under the influence of the highest volume of study sera was marginally higher than that of the corresponding volume of control sera; however, statistically the difference was not quite significant (*P* = 0.051).

#### Granulosa/theca cell competency to respond to FSH/LH

**Figure 2:**
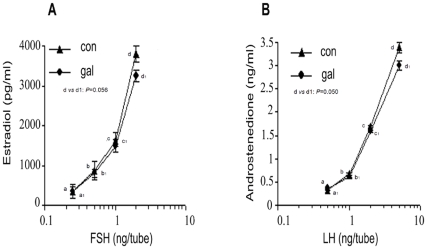
Granulosa and theca cell steroidogenic response to standard gonadotrophins. Granulosa and theca cells retrieved from both control and galactose-exposed rats were cultured in the presence of standard gonadotrophins. Steroidogenic response was evaluated with respect to FSH-induced granulosa cell production of E_2_ (Fig. 2A) and LH-induced theca cell production of androstenedione (Fig. 2B). Data points represent the mean ± SEM of 4 similar cultures. The figures show gradual increase in the production of E_2_ as well as androstenedione in the control and study groups in response to increase in the levels of FSH and LH, respectively, and the rate of increase does not differ significantly between the groups.

Figures 2A and 2B present the comparative degree of FSH-stimulated E_2_ and LH-stimulated androstenedione production, respectively by granulosa and theca cells collected from both control and study groups. In both groups, there was gradual rise in granulosa cell production of E_2_ or theca cell production of androstenedione in response to increments in the FSH and LH doses, respectively; and the rates of increase were statistically comparable between the groups. Thus granulosa and theca cell competencies to respond respectively to FSH and LH are not altered in galactose-treated rats.

#### Ovarian follicular atresia

**Figure 3:**
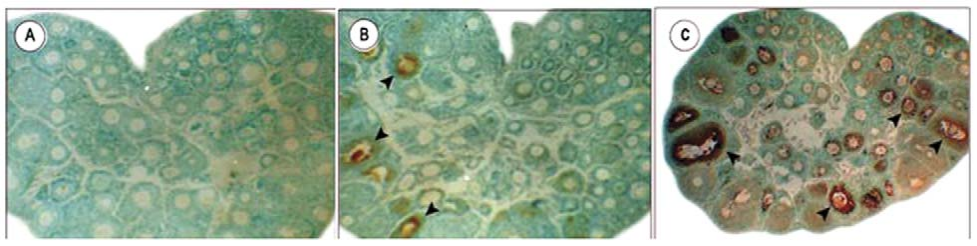
Immuno-histochemical detection of ovarian apoptosis by TUNEL. Ovarian apoptosis was evaluated by TDT-mediated dUTP nick-end labeling in representative sections from control (Fig. 3B) and galactose-exposed rats (Fig. 3C). The galactose-exposed rat ovaries show higher population of TUNEL sensitive cells as compared to controls. Negative control section (exclusion of terminal TdT) demonstrates no TUNEL reaction (Fig. 3A).

Immunohistochemical detection of follicular apoptosis by TUNEL demonstrated that the rate of follicular atresia in the galactose-exposed rat ovaries (Fig. 3C) increased as compared to the control group (Fig. 3B), which showed significantly lower TUNEL labeling. Negative control sections (exclusion of terminal TdT) demonstrated no TUNEL reaction (Fig. 3A).

### Effects of galactose *in vitro*

#### Follicular histoarchitecture

**Figure 4:**
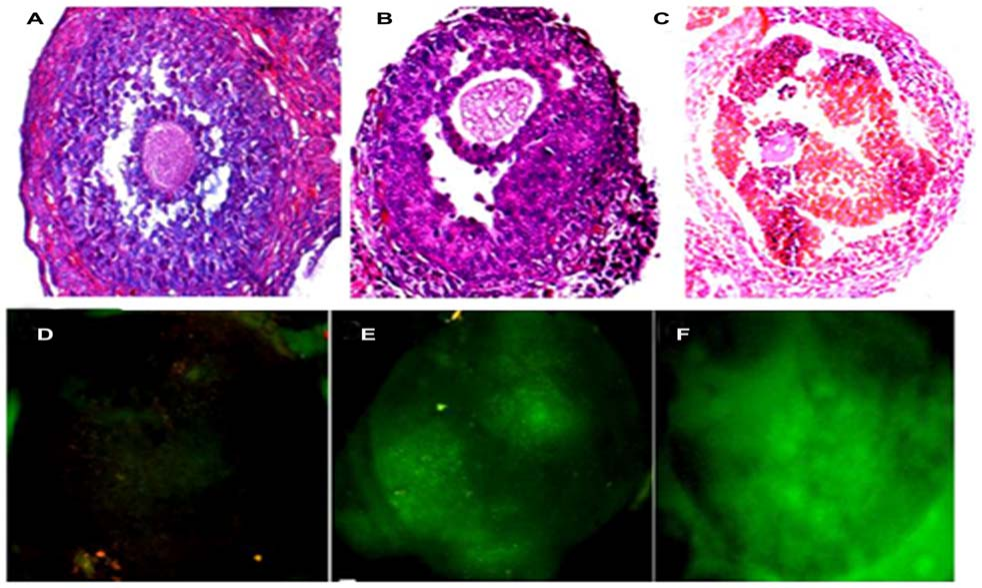
Histological assessment of follicular architecture and evaluation of follicular ROS generation. Histological pictures demonstrate that the follicles treated with galactose at 50 nM (Fig. 4B) and 100 nM (Fig. 4C) concentrations exhibit large number of atretic cells as compared to the untreated one (Fig. 4A). Follicles exposed to galactose at 50 nM (Fig. 4E) and 100 nM (Fig. 4F) concentrations show dose-dependent increase in intracellular ROS generation over that of the untreated control (Fig. 4D), as evaluated by fluorescence microscopy.

As compared to PBS-treated control ones (Fig. 4A), follicles exposed to galactose at 50 nM (Fig. 4B) and 100 nM (Fig. 4C) concentrations exhibited large number of pyknotic granulosa cells arranged in asymmetric rings that mark follicular degeneration [23].

#### Follicular ROS

Galactose treatment at 50 nM (Fig. 4E) and 100 nM (Fig. 4F) concentrations led to graded increase in ROS generation over that of the untreated cultured follicles (Fig. 4D).

#### Follicular caspase 3

**Figure 5:**
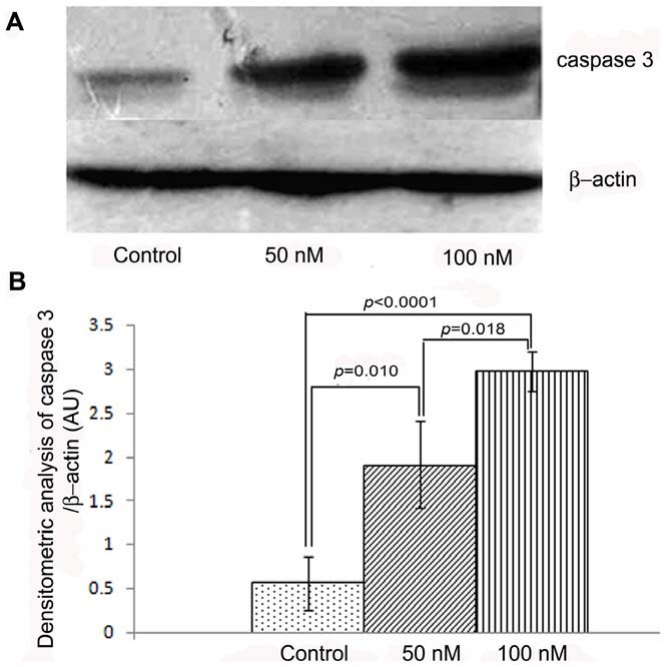
Immunoblot and densitometric analysis of caspase 3. Representative immunoblots of caspase 3 protein expressions in follicles cultured in the absence (control) and presence of galactose show dose-dependent increase in the expressions of cleaved form of caspase 3 protein in comparison to the untreated one (Fig. 5A). Both bands of caspase 3 were used for quantification. Compared with controls, the level of caspase 3 protein relative to β-actin increased significantly following treatment with galactose at 50 nM (*p* = 0.010) and 100 nM (*p*<0.0001) concentrations (Fig. 5B).

Immunoblot data showed dose-dependent increase in the expression of the apoptosis-executor protein, caspase 3, following treatment with galactose (Fig. 5A). The level of caspase 3 protein relative to β-actin increased significantly following treatment with galactose at 50 nM (*p* = 0.010) and 100 nM (*p*<0.0001) concentrations over that of PBS-exposed controls (Fig. 5B).

#### Granulosa cell mitochondrial membrane potential

**Figure 6:**
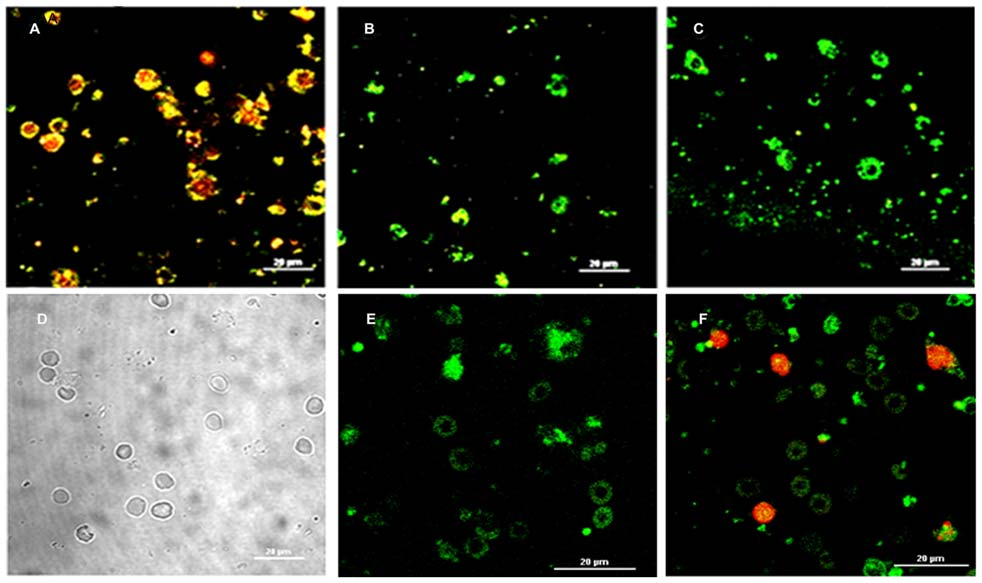
Potential-dependent mitochondrial JC1 staining and evaluation of apoptosis with annexin V. Images of mitochondrial JC-1 fluorescence in granulosa cells are presented in Figs. 6 A–C. The granulosa cells were cultured for 24 h in the presence of PBS (control) or galactose followed by incubation with JC-1 for 30 min at 37°C in dark. Cells were washed, fixed, and analyzed by confocal microscopy. The PBS-treated control group (Fig. 6A) shows greenish orange florescence in most of the cells due to strong JC-1 aggregation that marks high mitochondrial membrane potential. The cells treated with galactose at 50 nM (Fig. 6B) and 100 nM (Fig. 6C) concentrations show gradual shifting from greenish orange to green fluorescence indicating disruption of mitochondrial membrane potential. Merged images of annexin V and propidium iodide (PI) fluorescence in granulosa cells are presented in Figs. 6 D–F. The granulosa cells were cultured on poly L-lysine coated coverslips for 24 h in the presence of PBS (control) or galactose, followed by incubation with Annexin V-FITC and propidium iodide for 15 min at room temperature in dark. Cells were washed, fixed and visualized under confocal microscope. The PBS-treated control cells (Fig. 6D) show no annexin V binding. The cells treated with 50 nM galactose (Fig. 6E) show annexin V binding but no PI fluorescence, which mark the exposure of phosphadidylserine at the outer leaflet with intact membrane integrity. That the cells are apoptotic but not dead is indicated by the absence of red fluorescence of PI. The cells exposed to 100 nM galactose (Fig. 6F), by contrast, are clearly positive for both annexin V and PI fluorescence, which is indicative of loss of membrane integrity characterizing apoptotic death.

The granulosa cells exposed to PBS or galactose at concentrations up to 25 nM concentration exhibited high mitochondrial polarization as indicated by greenish orange fluorescence that marked JC-1 aggregation [only the PBS-exposed control picture (Fig. 6A) is shown]. But at concentrations 50 nM (Fig. 6B) and 100 nM (Fig. 6C), galactose gradually increased depolarization of mitochondrial membrane as evidenced by change from greenish orange fluorescence of JC-1 aggregates to green fluorescence of JC-1 monomers.

#### Granulosa cell annexin V-affinity assay

Figures 6 D–F present the merged images of annexin V and propidium iodide (PI) fluorescence of granulosa cells cultured in the presence of PBS (control) or galactose. Following exposure to galactose up to 25 nM concentration, the treated granulosa cells (not shown in the figure), like the controls (Fig. 6D), showed no annexin V binding. At 50 nM concentration of galactose (Fig. 6E), however, the cells showed characteristic annexin V binding without PI staining, indicating the intactness of plasma membrane. But at 100 nM galactose concentration (Fig. 6E) the granulosa cells exhibited both annexin V and PI fluorescence.

#### Granulosa cell p53 expression

**Figure 7:**
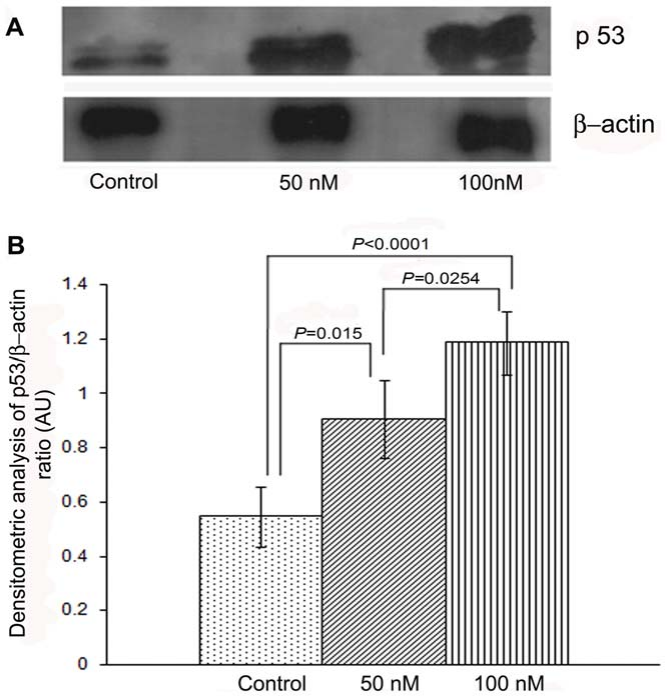
Immunoblot and densitometric analysis of p53. A representative immunoblot of p53 protein expression in cultured granulosa cells shows dose-dependent increase in its expression of p53 in the galactose-treated (50 nM; 100 nM) cells over that of the control (Fig. 7A). The histogram represents the relative intensity of the bands normalized to loading control. Quantification of p53 Western blots was done by the ratio of p53 to β-actin. Both bands of p53 doublet were used for quantification. Compared with controls, the expression of p53 protein relative to β-actin increased significantly following treatment with galactose at 50 nM (*p* = 0.015) and 100 nM (*p*<0.0001) concentrations (Fig. 7B).

Immunoblot analysis of total p53 showed that the protein ran as a doublet and there were relatively increased expressions in the galactose-treated granulosa cells (Figure. 7A). Compared with PBS-exposed controls, the p53 protein expression relative to β-actin increased significantly following treatment with galactose at 50 nM (*p* = 0.015) and 100 nM (*p*<0.0001) concentrations (Fig. 7B).

#### Preventive effects of E_2_ and FSH on granulosa cell expression of p53

**Figure 8:**
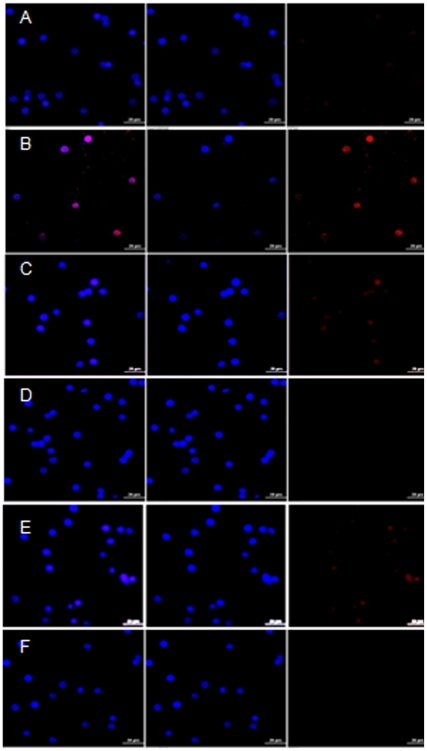
Reversal of galactose-induced granulosa cell p53 expression by co-treatment with E_2_ and FSH. Figure 8 demonstrates galactose effects on the granulosa cell expressions of p53 in the presence or absence of E_2_ and FSH. The granulosa cells were cultured for 24 h with PBS (control) or 50 nM galactose in the presence or absence of E_2_ and FSH, followed by overnight incubation with p53 antibody at 4°C. Cells were washed, incubated with secondary antibody, fixed, and analyzed by confocal microscopy. Galactose exposure (Fig. 8B) up-regulates granulosa cell p53 expression over that of untreated control (Fig. 8A). Co-treatment with 100 pg/ml E_2_ (Fig. 8C) or 25 ng/ml FSH (Fig. 8E) partially reversed the galactose-induced expression of p53, while co-treatment with E_2_ at 1 ng/ml concentration (Fig. 8D) or FSH at 100 ng/ml concentration (Fig. 8F) reversed the p53 expression back to control level (Fig. 8A).

Immunofluroscence detection of granulosa cell p53 expression by confocal microscopy revealed that the treatment of granulosa cells with 50 nM galactose (Fig. 8B) for 24 h increased the expression of p53 over that of untreated controls (Fig. 8A). Co-treatments with either E_2_ at 100 pg/ml (Fig. 8C) and 1 ng/ml (Fig. 8D) concentrations, or FSH at 25 ng/ml (Fig. 8E) and 100 ng/ml (Fig. 8F) concentrations prevented galactose-stimulated p53 expressions in a dose-dependent manner.

## Discussion

The precise mechanisms underlying impairment of ovarian function in classical galactosaemia are not known; nevertheless galactose and its metabolites are thought to play contributory roles at the ovarian level. The present study demonstrates that galactose toxicity attenuates FSH bioactivity and exerts direct ovotoxic effects.

In order to address the ovotoxic effects of Galactose, we adopted both *in vivo* and *in vitro* approaches. The *in vivo* effects of galactose toxicity were investigated in a previously described rat model that exhibited elevated blood levels of galactose and galactose-1-phosphate accompanied by characteristic phenotypes of POI including elevated serum gonadotrophins, resistance to gonadotrophins and delayed onset of puberty [13]. In our rat colony the mean age at onset of puberty is post-natal day (PND) 37. The present evaluations were therefore performed on PND 35, when the execution of the toxic effects of galactose is expected to be fully operational.

Since *in vivo* studies do not allow us to judge whether the ovarian effects of galactose are executed directly at the ovarian level or through systemic route, we performed some investigations *in vitro*. This was done in agreement with the animal ethics guidelines that recommended the use of minimal number of rats to the possible extent. Because of deficient follicular reserve, retrieval of necessary number of follicles/follicular cells from rats treated with galactose *in vivo* would have required large number of experimental rats, while for *in vitro* studies we could retrieve follicles/granulosa cells from 35-day old control rats that had good follicular yield because of optimum follicular reserve.

Our first approach was to evaluate the possibility of galactosylation defect under galactose toxicity. We followed the method adopted by Prestoz *et al.* [18] that determined the extent of galactose-acceptance capacity of serum glycoproteins *in vitro* in the presence of GalTase and sufficient UDP-galactose, which served as the galactose donor. Normally galactose is linked to N-acetyl glucosamine (GlcNAc) in the carbohydrate moieties of glycoproteins. The rationale for this approach was that any interference with the process of galactosylation would result in a greater number of glycoprotein oligosaccharides being terminated in GlcNAc. This would have increased capacity to accept galactose from radiolabeled UDP-galactose in the presence of purified GalTase. We observed a greater magnitude of galactose incorporation in the study sera, which is suggestive of a greater number of unoccupied galactose acceptor sites in the sera proteins produced under galactose toxicity. It may however be mentioned that although the culture was conducted in the presence of 1 µg of purified GalTase, the catalyzing reaction might have also been influenced by the endogenous serum GalTase activity. But it is significant to note that the increased serum binding of galactose in the study group occurred despite then having lower endogenous GalTase activity than control sera (described in subsequent section).

The enzyme GalTase catalyzes transfer of galactose from UDP-galactose to the terminal GlcNAc residues on the oligosaccharide complex in the process of galactosylation. Galactosaemia is associated with accumulation of galactose-1-phosphate and deficient production of UDP-galactose [24]. By measuring liver epimerase activity, we have earlier provided evidence for deficient UDP-galactose synthesis in rats under experimental galactose toxicity [13]. There is a report that accumulated galactose-1-phosphate may have inhibitory impact on GalTase activity [25]. To address the issue if galactose toxicity interferes with GalTase activity, we evaluated serum GalTase activity with respect to its capacity to catalyze the transfer of labeled galactose from UDP-galactose to ovalbumin. Reports indicate that irrespective of tissue origin, any significant alteration in GalTase activity is reflected in serum GalTase levels [26]. This remains the basis of assessing GalTase level in serum, instead of pituitary, which is the site of glycoprotein hormone (FSH/LH) synthesis. The present investigation demonstrated that the sera from galactose-exposed group had comparatively decreased capacity to catalyze the transfer of galactose from labeled UDP-galactose to the accessible GlcNAc terminal of ovalbumin under optimized culture condition. Taking the earlier report [13] and present findings together, a consensus can be made that restricted availability of UDP-galactose and attenuated GalTase activity perhaps rate-limit the process of galactosylation under galactose toxicity.

Gonadotrophins are members of the glycoprotein family of hormones. Carbohydrates attached to the protein core of these hormones influence a number of intracellular and extracellular processes including activation of the respective receptors and efficient triggering of signal transduction. It therefore appears logical to envisage a critical impact of galactose toxicity and consequent attenuation of galactosylation process on the biological activity of gonadotrophins.

Galactose toxicity increases serum levels of FSH and LH as measured by immunoassay [13]. As a measure of FSH-LH bioactivity, we evaluated serum gonadotrophic activity *in vitro*, which was based on the capacity of serum to stimulate steroidogenesis by granulosa and theca cells of control origin. The volumes of sera were selected in such a manner that they represented gonadotrophins at levels not exceeding the physiological range but were capable of stimulating E_2_/androstenedione production at concentrations within the detectable range of our assay system. The selection of sera volumes was therefore based on the pre-optimized doses of reference gonadotrophins that were shown to elicit measurable steroidogenic response in our culture set-up and the physiological range of serum gonadotrophins in rats [13]. The culture studies clearly demonstrated that sera from both control and galactose-exposed rats were almost equipotent in stimulating theca cell production of androstenedione (Figure 1B). Marginally increased rate of androstenedione production by the sera of galactose-exposed rats over that of respective volume of control sera might be attributed to elevated serum LH levels under galactose toxicity [13]. By contrast, despite having elevated levels of FSH [13], the study sera hardly exhibited any FSH-like bioactivity (Figure 1A). Galactose represents an important constituent of the carbohydrate side chain of FSH but not of LH [27]. So the observations taken together suggest that galactose-deficient variant(s) of FSH that lack bioactivity are perhaps produced under galactose toxicity.

Glycosylation defect in galactosaemia was first suggested by Haberland *et al.* [28] and further attested by many investigations [18], [29]–[35]. The loss of bioactivity following deglycosylation of FSH (27) and weaker FSH bioactivity in galactosaemic women [18] are in good agreement with each other. The present findings are therefore reminiscent of the earlier studies [18], [28]–[35] linking deficient galactosylation and attenuated biopotency of FSH. But reports that demonstrate no significant differences in either FSH isoforms [20] or FSH bioactivity [21] in the galactosaemia patients are contrary to these observations. By using Chinese hamster cells transfected to express human FSH receptors that produce cAMP in response to activation by FSH, Sanders *et al.* [21] demonstrated no loss of FSH bioactivity in galactosaemic women. No definite explanation can be put forward to explain this discrepant finding on FSH bioactivity in human galactosaemia and experimental galactose toxicity. However, it may be important to note that in normal women circulating FSH bioactivity is associated with isoforms with different oligosaccharide structures [36], and changes in gonadotrophin isoform occur through the different phases of menstrual cycle [37]. Thus FSH bioactivity significantly differs between the different phases of the same cycle, with highest bioactivity at mid-cycle [37]. Sanders and co-workers [21] were not particular about the phase of the menstrual cycle of the control subjects, when the blood was drawn for the assessment of FSH bioactivity. The finding of comparable FSH bioactivity between the control and galactosaemia women might not be highly unlikely if the control samples were collected during the luteal phase, when FSH bioactivity was the lowest [38]. However, further studies are needed to delineate the precise factors that account for the disparity in FSH bioactivities between the investigations.

It may be relevant to note in this context that unlike POI, testicular dysfunction is not a very frequent finding in human galactosaemia. Also, in a rat model for galactosaemia, Chen *et al.* demonstrated no corresponding testicular toxicity [39]. Differential ovarian and testicular resistance to galactosaemia is perhaps due to the facts that expression of GALT is lowest in testis [39] that makes this organ less vulnerable to galactose toxicity, and that FSH does not play an obligatory role in the maintenance of spermatogenesis [40] unlike in folliculogenesis.

Fraser *et al.* [22] suggested that perhaps an acquired anomaly of gonadotrophin receptors also attributes to the process of follicular resistance to gonadotrophins. We addressed the issue by evaluating steroidogenic response of the target follicular cells to reference gonadotrophin preparations. The results clearly demonstrate that both granulosa and theca cells from galactose-exposed ovary responded as effectively as the control follicular cells responded to FSH and LH to produce E_2_ and androstenedione, respectively (Figure 2 A & B). This observation refutes the proposition of receptor anomaly as the cause of follicular gonadotrophin resistance inducted by galactose toxicity.

It is indeed appropriate in this context to refer to our observation that the galactose-exposed rat ovaries that were normally refractory to gonadotrophin stimulation, responded well when endogenous gonadotrophins were suppressed by GnRH receptor down-regulation [13]. Earlier workers have demonstrated that deglycosylated FSH has higher binding affinity to its receptor than the glycosylated form, but is unable to activate second messenger system and thereby exert antagonistic effects at the receptor levels [19]. This very characteristic feature of deglycosylated forms of FSH explains why the ovary could respond favourably to exogenous gonadotrophins only after endogenous gonadotrophins were suppressed by pituitary desensitization [13].

Apoptotic death of follicles is an essential phenomenon in ovarian physiology occurring at all periods of life. That elevated galactose levels favour the activation of rat ovarian apoptosis has been substantiated earlier by the increased expression of apoptosis–mediating proteins and down-regulation of anti-apoptotic proteins [16]. The present observation on ovarian TUNEL reaction provides direct evidence in support of increased follicular apoptosis under experimental galactosaemic state.

Classical galactosaemia is characterized by deficiency of GALT and consequent intracellular accumulation of galactose, galactose-1-phosphate and galactitol. Two metabolites are potentially toxic: galactitol is responsible for the cataracts, while galactose-1-phosphate causes the rest of the pathology [41]. Ovary is the second richest organ for the expression and activity of GALT, and is therefore one of the most likely organs to accumulate galactose and galactose-1-phosphate in galactosaemia and get affected by their direct ovotoxic effects, if any [4]. We, therefore, examined the effects of galactose on isolated whole-follicle and granulosa cells *in vitro*.

To the best of our knowledge, there are no reports on the ovarian concentrations of galactose in human or experimental galactosaemia. To optimize the galactose doses for *in vitro* studies, initially we screened the effects of galactose on granulosa cell mitochondrial membrane potential and annexin-V affinity assays at concentrations ranging between 10–100 nM. Since no effects could be appreciated up to 25 nM concentration, we performed all culture studies in the presence of 50 nM and 100 nM galactose that are well below the blood galactose level (∼350 nM) under the present experimental situation [13].

In the whole-follicle culture, galactose dose-dependently increases follicular generation of ROS and caspase 3. This clearly suggests the direct atretic effects of galactose because ROS is the initiator of apoptotic cascade in granulosa cells [42], while caspase 3 is the key executor of all apoptotic processes [43]. A complex interactive network between the three follicular compartments - the oocyte, granulosa cells and theca cells - is operative in determining the fate of a follicle [44]. Reports indicate that follicular atresia is driven by the apoptosis of granulosa cells that secondarily leads to oocyte death. A number of intracellular molecules are possibly directly involved in the regulation of this process. GDF-9 is an oocyte-specific factor that plays a pivotal role in maintaining oocyte-granulosa-theca cell communications to promote follicular differentiation and maturation [45]. There is an earlier report that galactose significantly down regulates ovarian GDF-9 [15]; increased follicular atresia was considered a secondary consequence. We, however, observed that galactose can directly trigger granulosa cell apoptosis *in vitro*.

Viable cells maintain a strictly asymmetric lipid bilayer composition between the inner and outer leaflets of the plasma membrane, with phosphatadylserine (PS) residues at the cytoplasmic face [46]. Apoptotic cell death is accompanied by loss of phospholipid asymmetry in membrane structure by surface exposure of PS molecules at the outer membrane leaflet, while the membrane integrity remains unchallenged. Annexin V cannot penetrate viable cell membrane and therefore cannot bind PS, but it can bind with high affinity to the exposed PS in the presence of calcium [47]. Our confocal microscopic pictures of annexin V-affinity binding assay clearly demonstrate that following exposure to galactose at 50 nM concentration, the granulosa cell surfaces are annexin V-positive, but negative for staining by the membrane impermeable DNA stain PI. This indicates that galactose induces granulosa cell apoptosis. However, at 100 nM doses some cells showed PI fluorescence, which is indicative of cell death.

Reports suggest that early during apoptosis, cells undergo disruption of the mitochondrial transmembrane potential prior to PS exposure at the outer membrane leaflet [48]. JC-1 is a lipophilic cationic fluorescent dye that can enter selectively into mitochondria and act as a dual emission probe. As the membrane potential increases, JC-1 aggregates and changes its colour from green to orange, while it maintains the green colour of its monomeric form as the mitochondrial membrane potential depolarizes [49]. The confocal microscopic picture following JC-1 staining shows that galactose at 50 nM and higher concentrations disrupted the mitochondrial transmembrane potential. This finding also supplements the findings of annexin V binding.

Apoptosis can be activated either by endogenous intrinsic pathways resulting from an imbalance between pro-apoptotic and anti-apoptotic factors, or by exogenous extracellular mechanism [43]. Recent study documents that p53-mediated intrinsic death pathways is central in the induction of follicular atresia [50]. Our western blot and immunofluorescence analyses also showed that galactose dose-dependently up-regulated granulosa cell expression of p53 and disrupted mitochondrial membrane potential *in vitro*. FSH and E_2_ belong to the anti-apoptotic factors that significantly impact follicular survival [43]. The anti-apoptotic effect of FSH is mediated in part by suppression of ROS [42]. Since galactose toxicity attenuates FSH bioactivity and sets back granulosa cell production of E_2_, the resultant follicular microenvironment perhaps suffers from deficiency of two major anti-apoptotic factors. We hypothesize that in our experimental galactosaemia rat model, galactose can directly induce follicular oxidative stress, which is not taken care of by FSH because of its attenuated bioactivity. Consistent with this proposition are our findings that co-treatment with FSH or E_2_ effectively prevented galactose-induced granulosa cell expression of p53. This finding is important with respect to its therapeutic implications. It is pertinent in this respect to refer that several patients with classical galactosaemia and ovarian dysfunction responded to exogenous gonadotrophin administration, either by ovulating or by documented estrogen production [51], and pregnancy has been reported in galactosaemia patient after down-regulation of endogenous gonadotrophins followed by stimulation with recombinant FSH [52]. However, treatment with exogenous gonadotrophins is practically difficult to generalize since this can be a rational treatment option only for the subgroup of women with residual follicle reserve.

In conclusion, galactose exerts its ovotoxic effects perhaps at multiple levels. Ovarian accumulation of galactose and galactose-1-phosphate may exert direct apoptotic effects, while the extra-ovarian effect of galactose may involve attenuation of FSH bioactivity followed by withdrawal of protection from ROS insult and activation of p53-mediated granulosa cell apoptosis.

## Materials and Methods

### Chemicals and reagents

Most of the chemicals including D-galactose, bovine serum albumin (BSA), MnCl_2_, GalTase (from bovine milk), UDP-galactose, Adenosine 5^/^-monophosphate (5^/^ AMP), MES (2-[N-Morpholino] ethanesulphonic acid), trichloroacetic acid (TCA), ovalbumin, diethylstilbestrol (DES), McCoy's 5a medium (modified), Medium 199 (M 199) (with Earle's salts, L-glutamine and 25 mM HEPES), 5,5′,6,6′ tetrachloro-1,1′,3,3′-tetraethylbenzimidazolcarbocyanine iodide (JC-1), human chorionic gonadotrophin (hCG), haematoxylin, insulin, transferrin, linoleic acid, selenium, 2_, 7_-dichlorofluorescein diacetate, goat serum, and pregnant mare serum gonadotrophin were purchased from Sigma Chemical Co., St. Louis, MO, USA. Nutrient mixture HAM'S F-10 with L-glutamine (1.0 mM) and sodium bicarbonate (1.2 g/l) (Hyclone, Logan, Utah), Modified eagle's medium (Gibco™ Invitrogen corporation), Uridine diphospho-D-[6-^3^H]galactose, ammonium salt (Amersham Pharmacia Biotech UK Limited, Buckinghamshire, England), mounting medium for fluorescence (Vector laboratories Inc. Burlingame), TdT-FragEL™ DNA fragmentation kit (Oncogene Research Products, Cambridge, MA), RPMI 1640 media (Gibco, Grand Island, NY), eosin (s.d. fine-chem. Ltd, Mumbai, India), Poly-L-Lysine coated slides and four chambered slides (BD biosciences, Bedford, MA), Immobolin–P membranes (Millipore Crop, Billerica, MA), Annexin V-FITC apoptosis detection kit (BioVision, Mountain View, CA), immuno reagents for western blot analysis of p53 and β-actin (Santacruz Biotechnology, Santacruz, CA), secondary antibody Alexa 633 (Invitrogen Corporation, Carlsbad, CA) and super signal west pico chemiluminescent substrate (Thermo Scientific, Rockford, USA) were procured from the respective commercial sources. Rat FSH and LH reference preparations (rFSH-RP2 and rLH-RP3) were procured from National Hormone and Pituitary Programme, NIDDK, USA. Isocaloric food pellets (carbohydrate: 65.5%, protein: 21%, fat: 5.5%, mineral mixture: 7% and vitamin mixture: 1%), supplemented with or without 35% galactose, were prepared in the institute's animal house.

### Animals

The experiments were performed in accordance with the guidelines formulated by the Committee for the Purpose of Control and Supervision of Experiments on Animals, Ministry of Culture, India, with approval from the Animal Ethics Committee of Indian Institute of Chemical Biology (ID: 147/1999/CPCSEA/SNK-P8/08-08-2005). Pregnant Sprague-Dawley rats, procured from the random bred colony of the animal house of our institute, were maintained under good husbandry conditions supported by diurnal cycles of 12 h light and 12 h darkness with lights on at 0600 h daily. They were fed standard food pellet supplemented with or without 35% D-galactose from day 3 of conception continuing through weaning of the litters on postnatal day (PND) 21. On PND 35, few female pups from both control and treated groups were sacrificed, blood was collected by direct cardiac puncture and ovaries were dissected out. Sera were separated and evaluated for galactose incorporation capacity, GalTase activity, and gonadotrophin bioactivity, while ovaries were fixed and processed for the assessment of follicular atresia by TUNEL reaction. Other female litters from both groups received subcutaneous (sc) injection of DES (2 mg/rat/day) for 4 days and were sacrificed. Granulosa and theca cells were collected from the ovaries and cultured for the assessment of gonadotrophin receptor competency. The *in vitro* effects of galactose were assessed in whole follicles or isolated granulosa cells obtained from 35-day old control rats injected sc with DES (2 mg/rat/day) for 4 days, or PMSG (25 mIU/rat/day) for 2 days. The procedures are described in details under the heading of individual studies.

### *In vivo* studies

#### Incorporation of UDP-(^3^H) galactose into sera proteins

Sera from 35-day old rats from the galactose-exposed (n = 12) and control (n = 10) ones were tested for their capacity to incorporate galactose in the presence of radiolabeled UDP-[^3^H]galactose and commercially available GalTase, following the method of Ornstein *et al.* [33] with little modification. Each assay mixture (final volume 35 µl) contained 225 µg of serum protein (as determined by the method of Lowry *et al.* [53] using BSA as control), 50 mM/L MnCl_2_, 1 µg GalTase, 50 mmol/L MES and 15 mmol/L 5^/^ AMP at pH 6.5. The mixture was incubated at 37°C for 3 h. Enzyme activity was stopped thereafter and protein was precipitated by the addition of 10% TCA followed by centrifugation at 1200× g for 15 min. The pellet was washed twice in 0.1 N NaOH and finally counted in a Beckman LS-500 TD Liquid Scintillation Counter.

#### Assay of serum galactosyltransferase activity

Sera from control (n = 10) and galactose-exposed (n = 12) rats were evaluated for their relative GalTase activity by following the principle adopted for the galactose incorporation study; however, instead of using serum protein as the galactose acceptor and commercial GalTase as the catalytic enzyme, this assay utilized 235 µg of ovalbumin (150 µM) as the galactose acceptor [54] and serum as the source of GalTase.

#### Collection and culture of granulosa cells

The *in vitro* assays for FSH activity and its receptor competency were performed using an *in vitro* granulosa cell culture method developed by Jia and Hsueh [55] with modification [56] that uses aromatase activity in rat granulosa cells as an index of FSH bioactivity. The rats were treated with subcutaneous injection of DES (2 mg/rat/day) for 4 days [57]. Granulosa cells were released from their ovaries by puncturing follicles and cultured for 24 h at 37°C in a 5% CO_2_ incubator. Each well contained approximately 1×10^6^ viable cells in 0.5 ml of Ham's F10 medium, supplemented with L-glutamine (2 mM), BSA (100 mg/l), penicillin (100 IU/ml), streptomycin sulphate (100 µg/ml), 1-(3-isobutyl)-1-methylxanthine (0.125 mM), DES (10^−4^ M), insulin (1 µg/ml), hCG (100 µg/ml), and androstenedione (10^−6^ M) as the substrate for aromatase. The culture was conducted in the presence of FSH reference preparation and/or serum, as the situation demanded. The selected concentrations of FSH corresponded to the normal physiological range in the same age group [13]. On completion of incubation, the medium was aspirated and centrifuged. The resultant supernatants were stored frozen at −40°C until E_2_ was measured by fully automated chemiluminescence assay using specific E_2_ assay kit (ACS 180, Chiron Diagnostics Corp., East Walpole, MA, USA). The assay sensitivity was 10 pg/ml; both intra- and inter-assay coefficients of variation (COV) were <8%.

#### Examination of serum FSH activity

Granulosa cells (∼1×10^6^) of control ovarian origin were incubated in the presence of sera from control (n = 8) and galactose-exposed (n = 13) rats at four different volumes between 25 and 200 µl in a total incubation volume of 0.5 ml. Serum FSH activity was assessed with respect to its capacity to stimulate granulosa cell production of E_2_, expressed as picogram per ml culture medium, and plotted against each volume of serum to construct dose-response curves.

#### Examination of granulosa cell competency to respond to FSH

Granulosa cells were collected from ovaries of PND 35 control (n = 10) and galactose-exposed (n = 33) rats. Because of poor yield of cells in the study group, the granulosa cells were pooled from 3 rats for each determination. The cells were incubated in the presence of rat FSH reference preparation in the range between 0.25 and 2 ng per tube. The production of E_2_ in respect of each concentration of FSH was measured and a dose-response curve was plotted.

#### Collection, purification and culture of theca cells

The *in vitro* bioassays for LH and its receptor competency relied on androstenedione production by purified rat theca cells, prepared using the procedure described by Magoffin and Erickson [58]. Briefly, ovaries from DES-treated immature female rats were punctured gently in ice cold M199 medium, and theca cells were purified by an optimized discontinuous percoll density gradient centrifugation procedure, as described by Magoffin and Erickson [59]. Approximately ∼10^5^ purified rat theca cells were cultured in 500 µl of McCoy's 5a medium supplemented with L-glutamine (2 mM), penicillin (100 IU/ml) and streptomycin sulphate (100 µg/ml) for 48 h at 37°C in 5% CO_2_ in the presence of standard LH and/or serum samples. After incubation, the medium was collected and stored frozen at −40°C for subsequent analysis of androstenedione by an automated chemiluminescence assay system (Immulite 2000: Diagnostic Products Corp., USA). The assay sensitivity was 0.3 ng/ml, and intra-and inter-assay COV were 5.2% and 8%, respectively.

#### Examination of serum LH activity

Theca cells of control ovarian origin were incubated in the presence of sera from control (n = 8) and galactose-exposed (n = 13) rats at 3 different volumes between 50–200 µl. Androstenedione production rates were expressed as nanogram per ml.

#### Examination of theca cell competency to respond to LH

Theca cells collected from control (n = 9) and galactose-exposed (n = 42) rats were incubated in the presence of rat LH reference preparation in the range between 0.5–5.0 ng that corresponded to the normal physiological range of serum LH in the same age group [13]. Because of poor yield of cells in the study group, they were pooled from 3/4 rats. The production of androstenedione in respect of each concentration of LH was measured and a dose-response curve was plotted.

#### Effects of galactose on follicular atresia

Immunohistochemical detection of follicular apoptosis was done by *in situ* 3^/^-end labelling of DNA fragments in ovarian sections using the TdT-mediated dUTP nick-end labeling (TUNEL) assay according to the instructions given in the kit manual. Briefly, ovaries from control (n = 9) and galactose-exposed rats (n = 11) were immerse-fixed in 10% neutral buffered formalin at 4°C, dehydrated, embedded in paraffin, and serially sectioned at 5 µm thickness. Deparaffinized tissue sections were incubated with proteinase K (20 µg/ml) in a humidified chamber for 15 min and endogenous peroxidase activity was removed by treatment with 3% H_2_O_2_ for 10 min. Sections were then incubated with TdT labeling buffer at 37°C for 1 hr in a moist chamber. They were stained with diaminobenzidine as a peroxidase substrate and counterstained with methyl green. For control experiments, the enzyme incubation step was omitted. Reaction was documented by digital photography.

### *In vitro* Studies

#### Follicle culture

The immature female rats were injected subcutaneously with 10 IU of PMSG in 0.1 ml sterile 0.9% saline [42]. After 24 h the animals were euthanized, ovaries were removed, and the follicles at early antral stage of maturity and measuring 150–200 µm in diameter were dissected out. Without any assessment of oocyte maturity, the follicles were cultured by the method previously described by McGee *et al.* [60]. Briefly, the culture was continued for 24 h in Modified Eagle's Medium supplemented with ITS1 (insulin, 10 mg/L; transferrin, 5.5 mg/L; linoleic acid, 4.7 mg/L; selenium, 5 mg/L) and Pen/Strep (penicillin, 100 U/ml; streptomycin, 100 mg/ml) in the presence of PBS or galactose (50 nM and 100 nM) and overlaid with sterile mineral oil at 37°C in a moist atmosphere of 5% CO_2_ and 95% air. On completion of incubation the follicles were collected for the following analyses.

#### Histological analysis of follicles

Follicles were fixed in 4% paraformaldehyde solution, embedded in paraffin and sectioned (5 µm). Sections were stained with haematoxylin and eosin and examined under light microscope. Eight to ten follicles from each treatment group were analyzed, and 1 representative follicle from each group was photographed.

#### Detection of follicular ROS generation *in situ*

Follicular generation of ROS was evaluated according to Turton *et al* [42] with little modifications. Briefly, the control and galactose-treated follicles were washed with PBS and incubated with 100 µm 2_, 7_-dichlorofluorescein diacetate (H_2_DCFDA) in MEM media for 30 min. ROS-mediated oxidative transformation of H_2_DCFDA to fluorescent dichlorofluorescein was evaluated by fluorescence microscopy as the measure of ROS.

#### Western blot analysis of follicular caspase 3

Twenty follicles from the control and each treatment groups (50 nM and 100 nM) were collected in Eppendorf tubes in lysis buffer (50 mM Tris-HCl, 150 mM NaCl, 1% SDS, 5 mM EGTA, 0.5 mM MgCl_2_, 0.5 mM MnCl_2_ and 0.2 mM phenylmethylsulfonylfluoride supplemented with protease inhibitor). These were homogenized with a glass rod and centrifuged at 7500 rpm for 10 min at 4°C. The supernatant was collected and estimated for protein concentration. Fifty microgram of total protein from each sample was resolved on a 10% sodium dodecyl sulfate polyacrylamide gel and transferred onto immobilon-P membranes. The membrane was incubated with 5% blocking solution (Tris-buffered saline [TBS] containing 0.1% Tween-20, 5% non-fat dried milk) for 2 h, washed with TBS containing 0.1% Tween-20, and incubated overnight with rabbit polyclonal anti-caspase 3 antibody (1∶800) and anti-β-actin-antibody (1∶1000). HRP-conjugated secondary mouse anti-rabbit antibody (1∶2000) was added and peroxide activity was visualized by enhanced chemiluminescence and exposed to X-Ray film. Densitometric quantification of signals was done by Image-J software. The data were expressed as caspase 3 to β-actin ratio.

#### Granulosa cell isolation and preparation

The ovarian granulosa cells from DES-treated control immature rats were collected in ice-cold PBS, centrifuged, washed, and re-suspended in RPMI medium. The cells were cultured on poly L-lysine-coated microscopic glass slides in the presence of PBS or galactose at concentrations ranging from 10 nM to 100 nM in humidified atmosphere containing 5% CO_2_ and 95% air at 37°C for 24 hours, and analyzed for mitochondrial membrane potential, annexin V-affinity binding, and western blot analysis of p53. The cell viability was more than 90% in all sets of experiments, as measured by trypan blue dye exclusion test.

#### Detection of granulosa cell mitochondrial membrane potential

Analysis of mitochondrial membrane potential was done by staining with JC-1, a lipophilic cationic fluorescent dye capable of selectively entering mitochondria and acting as a dual emission probe that reversibly changes color from green (FL-1) to greenish orange (FL-2) in concert with polarization of mitochondrial membrane [61]. On completion of culture for 24 h, the granulosa cells were incubated with JC-1 for 30 min at 37°C in darkness. Cells were washed with PBS. The cover slips were inverted on glass slides, fixed in 2% formaldehyde, and analyzed by confocal microscopy (Nikon, A1R, Japan).

#### Granulosa cell annexin V binding assay

The cultured granulosa cells on the poly L-lysine coated coverslips were flooded with 500 µl of 1X binding buffer, 5 µL of Annexin V-FITC, and 5 µL of propidium iodide (PI), and incubated at room temperature for 15 min in dark. The cover slips were inverted on glass slides, fixed in 2% formaldehyde, and visualized under confocal microscope.

#### Western blot analysis of granulosa cell p53

The cultured granulosa cells were lysed in buffer (150 mM NaCl, 500 mM Tris, 10 mM EDTA) supplemented with protease inhibitors (1 µg/ml aprotinin, 1 µg/ml pepstatin, 1 µg/ml leupeptin, 1 mM PMSF, 1 µg/ml trypsin inhibitor) and 1% Triton X-100, and centrifuged at 5,000 rpm for 10 min at 4°C. The supernatant was collected and estimated for protein concentration. Fifty microgram of total protein from each sample was resolved on a 10% sodium dodecyl sulfate polyacrylamide gel and transferred onto immobilon-P membranes. The membrane was incubated with 5% blocking solution (Tris-buffered saline [TBS] containing 0.1% Tween-20, 5% non-fat dried milk) for 2 h, washed twice with TBS containing 0.1% Tween-20, and incubated for 4 h with rabbit polyclonal anti-p53 antibody (1∶500 dilution) and anti-β-actin antibody (1∶1000). HRP-conjugated secondary mouse anti-rabbit antibody (1∶2000) was added and peroxide activity was visualized by enhanced chemiluminescence and exposure to X-Ray film. Densitometric quantification of signals was done by Image-J software. The data were expressed as p53 to β-actin ratio.

#### Immunofluorescence detection of granulosa cell p53 expression

Granulosa cells collected from immature control rat ovaries were grown on four-chambered slides and incubated for 24 h with 50 nM galactose in the presence or absence of E_2_ (100 pg/ml and 1 ng/ml) or FSH (25 ng/ml and 100 ng/ml). The cells were washed with PBS and fixed for 10 min in ice-cold absolute methanol at −20°C. The slides were dried, rinsed with PBS, and incubated with 10% normal goat serum in PBS for 30 min to suppress non specific binding. After removing the serum solution, the slides were incubated overnight at 4°C with mouse monoclonal p53 antibody (1∶200 dilution) in PBS containing 1.5% normal goat serum. The slides were washed and incubated for 2 h in dark with Alexa 633 anti-mouse secondary antibody (1∶400 dilution) in PBS containing 1.5% normal goat serum. Finally, the slides were thoroughly washed in PBS, mounted with DAPI-containing media, and observed under confocal microscope.

### Statistical analyses

The data were expressed as mean ± standard error of the mean (SEM), where ‘n’ refers to the number of animals or determinations. All treatments of the granulosa as well as theca cells in culture with either FSH/LH or serum samples were carried out in triplicate, and each experiment was repeated at least twice. Given that the numbers of determinations were low, two-tailed Student's *t*-test was used to analyse the significance of differences between the experimental and control observations. A difference was considered statistically significant at *P*<0.05.
